# Ligation Versus Repair of Isolated Traumatic Peripheral Venous Injury: A Systematic Review and Meta-Analysis

**DOI:** 10.3400/avd.ra.25-00107

**Published:** 2026-04-22

**Authors:** Noman Shahzad, Muhammad Asad Moosa, Nadeem Ahmed Siddiqui, Fareed Ahmed Shaikh

**Affiliations:** 1 Doncaster and Bassetlaw Teaching Hospitals NHS Foundation Trust, Doncaster, United Kingdom; 2 Worcester Royal Hospital, Worcester, United Kingdom; 3 Section of Vascular Surgery, Department of Surgery, Aga Khan University Hospital, Karachi, Pakistan

**Keywords:** traumatic venous injury, ligation, repairs

## Abstract

There are no guidelines to suggest repair versus ligation for traumatic deep venous injuries. We aimed to compare the outcomes after reconstruction versus ligation of isolated traumatic venous injuries. This systematic review and meta-analysis (PROSPERO Registration Number: CRD42019143136) included all literature reported on the management of isolated venous injuries in adult trauma patients, excluding case reports from 1950 to 2020. Primary outcomes were mortality and amputation, while compartment syndrome, chronic venous hypertension, deep venous thrombosis, and pulmonary embolism were the secondary outcomes. PubMed, Google Scholar, Cochrane database, and Web of Science were searched. Study selection and synthesis were done following Preferred Reporting Items for Systematic Reviews and Meta-Analyses (PRISMA) guidelines. A total of 25 studies were included. All the studies were observational, with the majority being retrospective in nature. Results of our meta-analysis show that ligation is significantly associated with higher rates of amputation (odds ratio [OR] = 1.73, 95% confidence interval [CI] = 1.20–2.48, p = 0.003) and mortality (OR = 1.50, 95% CI = 1.09–2.06, p = 0.01), whereas there is no significant difference in the rates of chronic venous insufficiency, deep venous thrombosis, and pulmonary embolism. There is insufficient data to analyze various types of venous repair, as well as to account for clinical severity at the time of presentation. Our results favor repair over ligation.

## Introduction

Traumatic vascular injuries can be life-threatening if not treated promptly and adequately. The reported incidence of mortality related to vascular trauma is around 9% globally.^1)^ The mortality rates are higher in low- and middle-income countries (LMICs) than in the developed nations.^2)^ Management of isolated venous injuries in trauma patients generally follows 2 principal strategies: ligation of the injured vein to achieve rapid hemorrhage control or venous reconstruction with the aim of preserving venous outflow and reducing long-term morbidity. Ligation is often expedient and well tolerated, particularly in unstable patients or when collateral venous drainage is adequate; however, it may be associated with limb edema and chronic venous insufficiency.^3,4)^

Venous repair may be undertaken by primary repair using nonabsorbable monofilament sutures when the defect is small and tension-free approximation is feasible.^4)^ Larger defects may require patch venoplasty, while extensive or segmental venous injuries necessitate interposition grafting to restore venous continuity.^5)^

Autologous vein is the preferred material for both patch repair and interposition grafting due to superior long-term patency, resistance to infection, and favorable hemodynamic properties.^5–7)^ However, harvesting autologous vein increases operative time and may require harvesting from an uninjured limb, thereby adding technical complexity. As an alternative, prosthetic materials may be utilized, including biologic grafts such as bovine pericardium or synthetic conduits such as Dacron or expanded polytetrafluoroethylene (ePTFE). While these materials reduce operative time and avoid vein harvest, available evidence suggests inferior long-term patency compared with autologous vein, particularly in contaminated fields.^5–7)^

There has always been debate on how to manage peripheral venous injuries, as repair has not been shown to have a clear advantage or increased risk over ligation. Much of the earlier literature comes from warfare experiences. Although repair was attempted whenever possible since the start of the 19th century, many patients with venous injuries were managed successfully by ligation of the injured vein during World Wars I and II.^8)^ However, in the Korean War in the 1950s, it was noticed that venous ligation led to significant limb edema secondary to venous hypertension and even led to limb loss from venous gangrene.^9)^ Later, similar results were reported by Rich and Hughes from the experience of the Vietnam War.^10)^ They analyzed data from the Vietnam War registry and advocated the benefits of venous reconstruction, with good short- and long-term outcomes.^10)^

Despite the limb salvage advantage of repair and reconstruction demonstrated in literature from warfare experiences, later studies from civilian injuries failed to replicate the benefits of repair. Furthermore, the risk of venous thrombosis and hence pulmonary embolism after repair led to a significant increase in morbidity and mortality.^11–13)^ Given the lack of clear advantage of repair versus the risks, and the quickness of venous ligation, many vascular surgeons advocate ligation of veins with fasciotomy, especially in trauma patients who are hemodynamically unstable.

### Objective

This study aimed to compare the morbidity and mortality after venous reconstruction versus ligation of isolated peripheral deep venous injuries in patients with trauma.

## Methodology

### Eligibility criteria

We included literature on the management of isolated peripheral venous injuries in adult trauma patients. Observational and interventional studies, including case series, cross-sectional, case–control, cohort studies, and clinical trials, were included, while case reports and review articles were excluded. Studies performed on animals were also excluded. We included only published literature in the English language from January 1950 to June 2022.

### Intervention

Two alternative interventions investigated were repair versus ligation of the peripheral deep veins.

Repair: The injured peripheral deep vein is repaired, either primarily by suturing the injured ends together or by using autologous or artificial graft, where the tissue defect is extensive and does not allow primary repair.

Ligation: The ends of the injured vein are ligated. It is sometimes accompanied by distal fasciotomy.

### Primary outcomes

Mortality: Death within 30 days of the procedure.

Amputation: Amputation of the limb after intervention for isolated deep venous injury within 30 days of the procedure.

### Secondary outcomes

Secondary outcomes were compartment syndrome, chronic venous hypertension, deep venous thrombosis, and pulmonary embolism.

### Information sources

We performed a thorough systematic search in the following search engines to identify relevant studies:

- Google Scholar (https://scholar.google.com)
- PubMed (http://www.ncbi.nlm.nih.gov/pubmed)
- Cochrane Database (http://onlinelibrary.wiley.com/cochranelibrary/search)
- EBSCO CINAHL Plus
- Web of Science

### Search strategy

A systematic search was conducted to identify relevant studies that met the selection criteria. The search strategy was developed to identify studies that included the population, intervention, and outcome of interest (Population/Problem, Intervention, Comparison, and Outcome [PICO] model). To rigorously search for each category, search terms were identified as follows.

### Population

Terms used to identify the population of interest: “Vascular Trauma,” “Venous Trauma,” “Vascular Injury*,” “Venous Injury*,” and “Isolated Venous Injury.”

### Intervention terms

Terms used to identify the intervention of interest are: “Repair,” “Reconstruction,” “Ligation,” and “Anastomosis.”

### Outcome

Terms used to identify outcomes of interest are: “Venous Hypertension,” “Oedema,” “Compartment Syndrome,” “Fasciotomy,” “Amputation,” “Venous Thrombosis,” “Pulmonary Embolism,” “Patency,” “Post-Thrombotic Syndrome,” “Death,” and “Mortality.”

Terms from each concept were searched by combining with “OR,” while the 3 concepts were combined by “AND” to rigorously identify all studies that met the selection criteria.

### Study selection

Study selection was done in a systematic manner. Two investigators performed an independent search to systematically identify all relevant studies. In case of discrepancies, a third investigator was involved to decide on inclusion of a particular study in the systematic review. Initial identification of studies was done by running the search strategy on all included search engines, and all relevant studies were identified. Duplicates and irrelevant studies were next identified by reviewing titles and abstracts. In the final stage, full manuscripts were reviewed. References of the included studies were also explored to identify any missing literature.

### Statistical analysis

Outcome summary measures from all studies are reported as frequencies, proportions, and percentages for individual studies, as well as for cumulative data.

RevMan 5 software was used for meta-analysis,^14)^ and results are reported in forest plots.

Variability among studies’ outcomes included in the meta-analysis was assessed using Cochran’s Q test and I^2^ values. In cases of significant variability on Cochran’s Q test or an I^2^ value greater than 50%, a random-effects model was used, whereas a fixed-effects model was used if variability was not significant on Cochran’s Q test and the I^2^ value was less than or equal to 50%.

### Risk of bias assessment

The possibility of reporting bias was assessed by visual inspection of a funnel plot for the primary outcome.

## Results

**Fig. 1:**
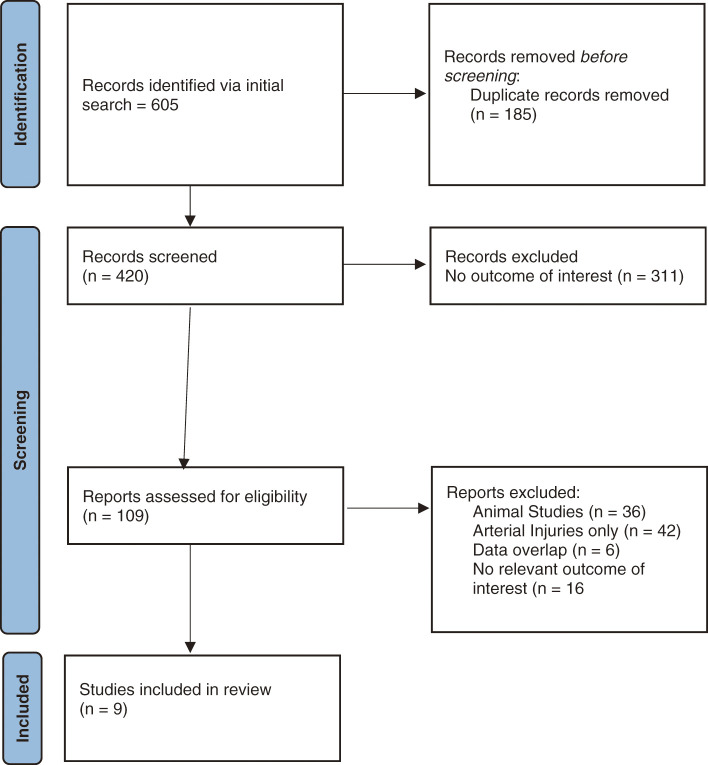
PRISMA flow diagram. PRISMA: Preferred Reporting Items for Systematic Reviews and Meta-Analyses

The initial search retrieved 605 studies, of which 185 were duplicates. Further exclusions were made for animal studies (36), arterial injuries (42), and no population or outcome of interest (311). Nine studies met the selection criteria and were included in the meta-analysis. The Preferred Reporting Items for Systematic Reviews and Meta-Analyses (PRISMA) diagram for study selection is shown in **Fig. 1**.

### Characteristics of included studies

All of the included studies were retrospective cohort. A total of 3090 patients were included, of whom 1418 underwent ligation and 1609 underwent repair. Details of the included studies are given in **Table 1**.

**Table 1:** Characteristics of included studies

Author	Year	Study design	Country	Repair total	Ligation total	Injury site
Megee et al.^28)^	2018	Ret. cohort	USA	226	192	Iliac vein
Matsumato et al.^29)^	2019	Ret. cohort	USA	1091	1029	Lower extremity vein from iliacs and below
Quan et al.^19)^	2008	Ret. cohort	USA	38	65	Lower extremity vein from iliacs and below
Manley et al.^30)^	2018	Ret. cohort	USA	49	35	Lower extremity vein from iliacs and below
Pappas et al.^20)^	1997	Ret. cohort	USA	73	23	Lower extremity vein from iliacs and below
Pasch et al.^31)^	1986	Ret. cohort	USA	41	22	Lower extremity vein from iliacs and below
Yelon and Scalea^12)^	1992	Ret. cohort	USA	31	48	Lower extremity vein from iliacs and below
Rich et al.^32)^	1976	Ret. cohort	USA	53	57	Pop vein
Blumoff et al.^33)^	1982	Ret. cohort	USA	7	10	Femoral veins

Ret.: retrospective

### Results of meta-analysis

#### Mortality

**Fig. 2:**
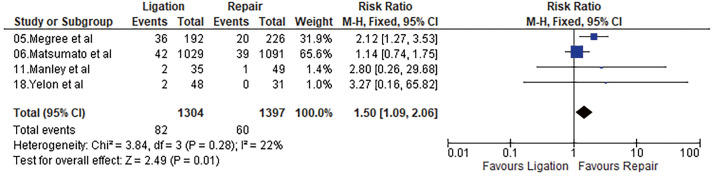
Forest plot of mortality. M–H: Mantel–Haenszel; CI: confidence interval; df: degrees of freedom

Four studies reported mortality within 30 days of the procedure. Overall mortality after repair of the injured vein was 4.29%, while it was 6.29% after ligation. Meta-analysis using a fixed-effects model showed a significant difference favoring repair (odds ratio [OR] = 1.50, 95% confidence interval [CI] = 1.09–2.06, p = 0.01, I^2^ = 22%). Results are shown in **Fig. 2**.

#### Major limb amputation

**Fig. 3:**
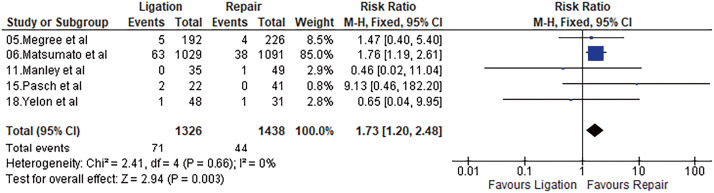
Forest plot of major amputation. M–H: Mantel–Haenszel; CI: confidence interval; df: degrees of freedom

Five studies reported major amputation within 30 days of the procedure. The major amputation rate was 3.06% after repair, while it was 5.35% after ligation. Meta-analysis using a fixed-effects model showed a significant difference favoring repair (OR = 1.73, 95% CI = 1.20–2.48, p = 0.003, I^2^ = 0%). Results are shown in **Fig. 3**.

#### Deep venous thrombosis

**Fig. 4:**
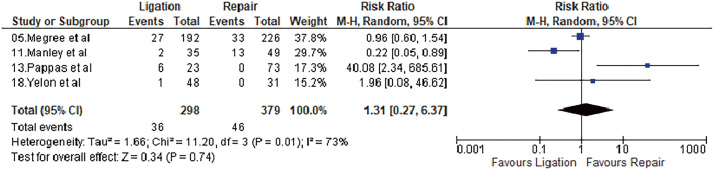
Forest plot of DVT. DVT: deep venous thrombosis; M–H: Mantel–Haenszel; CI: confidence interval; df: degrees of freedom

Four studies reported deep venous thrombosis after the procedure. It was 12.14% and 12.08% after repair and ligation, respectively. Meta-analysis using a random-effects model did not show a statistically significant difference (OR = 1.31, 95% CI = 0.27–6.37, p = 0.74, I^2^ = 73%). Results are shown in **Fig. 4**.

#### Pulmonary embolism

**Fig. 5:**
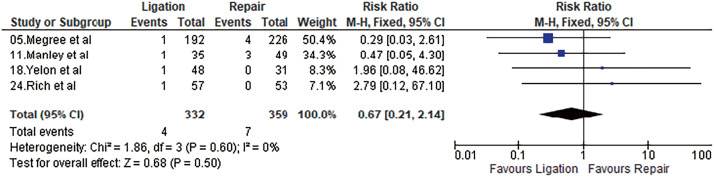
Forest plot of pulmonary embolism. M–H: Mantel–Haenszel; CI: confidence interval; df: degrees of freedom

Four studies reported pulmonary embolism after the procedure. It was 1.95% and 1.20% after repair and ligation, respectively. Meta-analysis using a fixed-effects model did not show a statistically significant difference (OR = 0.67, 95% CI = 0.21–2.14, p = 0.50, I^2^ = 0%). Results are shown in **Fig. 5**.

#### Limb swelling

**Fig. 6:**
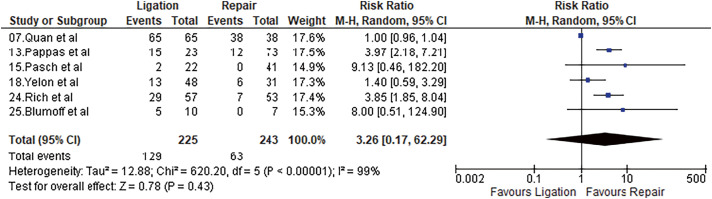
Forest plot of limb swelling. M–H: Mantel–Haenszel; CI: confidence interval; df: degrees of freedom

Six studies reported limb swelling after the procedure. It was 25.93% and 57.33% after repair and ligation, respectively. Meta-analysis using a random-effects model did not show a statistically significant difference (OR = 3.26, 95% CI = 0.17–62.29, p = 0.43, I^2^ = 99%). Results are shown in **Fig. 6**.

### Risk of publication bias

**Fig. 7:**
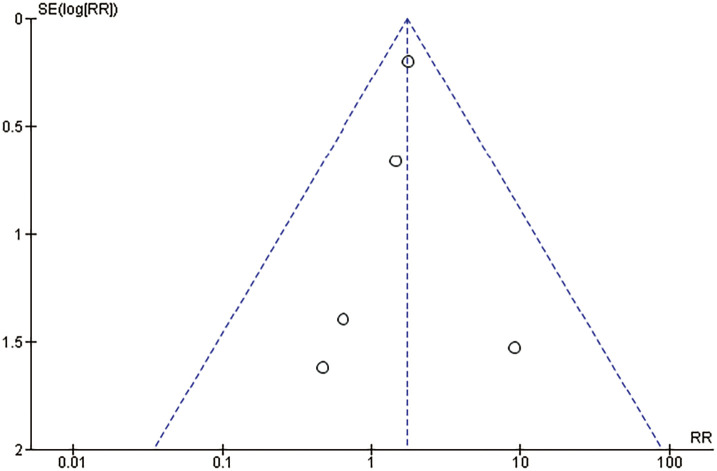
Funnel plot of major amputation. SE: standard error; RR: risk ratio

**Fig. 8:**
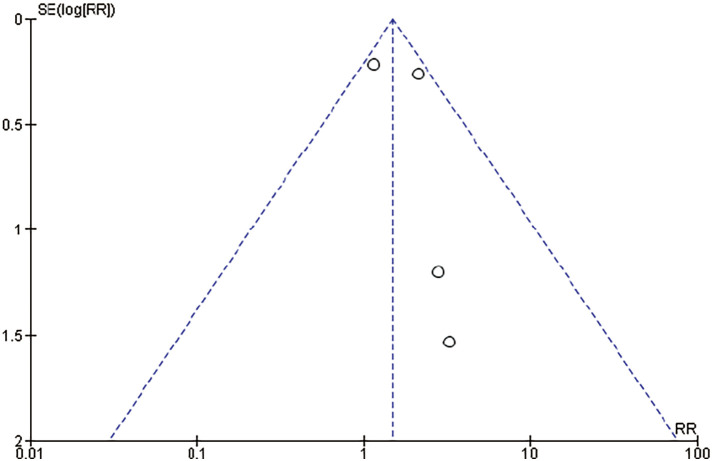
Funnel plot of mortality. SE: standard error; RR: risk ratio

Funnel plots for the primary outcomes of mortality and major limb amputation are shown in **Figs. 7** and **8**, respectively. No studies fell outside the 95% confidence limits, and included studies were distributed uniformly around the central vertical line, demonstrating low risk of publication bias.

## Discussion

Limited literature is available to guide evidence-based management of isolated deep venous injuries. Acute venous occlusion can lead to limb-threatening venous hypertension, venous ischemia, and even gangrene requiring major limb amputation.^15–17)^ For those who survive the acute episode, the risk of developing chronic venous hypertension and later sequelae of deep venous insufficiency is high.^18,19)^ Reconstruction of injured veins restores continuity of blood drainage distal to the injury, which has the advantage of preventing venous stasis and hypertension. However, the reconstruction procedure increases procedure time and complexity, which may not always be feasible. Furthermore, 30-day patency after primary repair or vein patch is reported to be 87%–88%, while 30-day patency rate is only 50% if a prosthetic graft is used for repair.^20)^ Prosthetic graft infection is also reported to be 1%–6% in elective settings^21,22)^; this number is likely to be high in trauma patients.

This meta-analysis shows that mortality and limb salvage after repair of isolated deep venous injuries of the lower limbs are superior to ligation. Venous ligation can exacerbate venous hypertension and outflow obstruction, increasing distal edema, tissue ischemia, and compartment pressures, thereby elevating the risk of fasciotomy and secondary amputation compared with repair, which preserves venous drainage.^23,24)^ Persistent venous hypertension may also prolong inflammatory responses and microvascular dysfunction, impairing limb perfusion. Additionally, loss of axial venous flow can augment reperfusion injury and tissue swelling, which contributes to worse limb outcomes. A meta-analysis by Byerly et al. also demonstrated higher amputation and mortality rates after ligation of inferior vena cava, suggesting that maintaining venous continuity mitigates these pathophysiological sequelae when technically feasible.^25)^

Our results did not show any statistically significant difference in deep venous thrombosis, pulmonary embolism, and limb swelling after repair versus ligation of the injured vein. The results are in line with the plausible explanation described above.

Although the results of the meta-analysis show that mortality and limb salvage outcomes of repair are superior to ligation, there are certain limitations to these results. All the studies were observational, and there are multiple factors that affect the decision-making of individual trauma patients. These include mechanism of injury,^26)^ concomitant musculoskeletal injuries, segment of the vein involved, presence of paired deep veins, concomitant other vascular injuries, time elapsed since trauma, salvageability of the limb upon presentation, clinical status of the patient, and available skills and resources.^27)^ We did not have enough data to evaluate and report on these factors. Furthermore, the extent of tissue injury and method of repair were not well described in many studies, which limits our ability to categorize outcomes according to the type of repair performed. Another limitation of this meta-analysis is the small number of studies available for analysis, which limits the generalizability of the study results.

This meta-analysis is one of the very first attempts to systematically analyze the available literature on outcomes of isolated venous injuries. Despite the favorable results of venous reconstruction shown in this meta-analysis, further well-designed prospective studies are needed to explore the management of venous injuries in trauma patients.

## Conclusion

Mortality and limb salvage rates after repair of isolated traumatic injuries to peripheral deep veins are better than those after ligation. Further high-quality studies are needed to validate these findings.
